# Protein Kinase C at the Crossroad of Mutations, Cancer, Targeted Therapy and Immune Response

**DOI:** 10.3390/biology12081047

**Published:** 2023-07-26

**Authors:** Angelo Aquino, Nicoletta Bianchi, Anna Terrazzan, Ornella Franzese

**Affiliations:** 1 Department of Systems Medicine, University of Rome Tor Vergata, 00133 Rome, Italy; angelo.aquino@uniroma2.it; 2 Department of Translational Medicine, University of Ferrara, 44121 Ferrara, Italy; nicoletta.bianchi@unife.it (N.B.); anna.terrazzan@unife.it (A.T.); 3 Laboratory for Advanced Therapy Technologies (LTTA), University of Ferrara, 44121 Ferrara, Italy

**Keywords:** PKC, PKC mutations, PKC isoforms, PKC inhibitors, cancer therapy, immune cells, immune checkpoint molecules, immune checkpoint inhibitor blockade

## Abstract

**Simple Summary:**

This review aims to analyze the clinical drawbacks faced by PKC inhibitors and uncovering aspects that might potentially interfere with the clinical outcome of compounds targeting PKC. In-depth analyses of the impact of key PKC mutations, the failures and promises of clinical trials conducted over the years and of the key immunological pathways involving distinctive isoforms in the tumor microenvironment, are critical to improve the drug targeting of PKC. We believe that a more inclusive view of the impact of different PKC variants on both cancer and immune cells should guide future efforts, which must consider current immunotherapy strategies. Also, a comprehensive representation of the pathways and partners involving PKC variants may help provide response biomarkers, which can indicate whether or which PKC isoform can be proficiently targeted, potentially in a feasible combination with immune checkpoint blockade.

**Abstract:**

The frequent PKC dysregulations observed in many tumors have made these enzymes natural targets for anticancer applications. Nevertheless, this considerable interest in the development of PKC modulators has not led to the expected therapeutic benefits, likely due to the complex biological activities regulated by PKC isoenzymes, often playing ambiguous and protective functions, further driven by the occurrence of mutations. The structure, regulation and functions of PKCs have been extensively covered in other publications. Herein, we focused on PKC alterations mostly associated with complete functional loss. We also addressed the modest yet encouraging results obtained targeting PKC in selected malignancies and the more frequent negative clinical outcomes. The reported observations advocate the need for more selective molecules and a better understanding of the involved pathways. Furthermore, we underlined the most relevant immune mechanisms controlled by PKC isoforms potentially impacting the immune checkpoint inhibitor blockade-mediated immune recovery. We believe that a comprehensive examination of the molecular features of the tumor microenvironment might improve clinical outcomes by tailoring PKC modulation. This approach can be further supported by the identification of potential response biomarkers, which may indicate patients who may benefit from the manipulation of distinctive PKC isoforms.

## 1. Introduction

The protein kinase C (PKC) family, first discovered in 1977 by Takai et al. [1], includes distinctive phospholipid-dependent enzymes able to phosphorylate the Ser and Thr residues of many substrates involved in either the promotion or suppression of cancer. PKC activation involves a sophisticated mechanism implicating its association with the cell membrane, followed by its priming and conformational modifications produced by the engagement with additional factors or second messengers. Three distinct groups of PKCs, encoded by nine genes, have been characterized based on specific control mechanisms and the second messenger involved in their activation. Conventional (c)PKCs include the isoforms α, βI, βII, and γ, and require diacylglycerol (DAG), Ca^2+^ and phosphatidylserine (PS) for their activation [2], while novel (n)PKCs, including the δ, ε, η and θ isotypes, require only DAG [3]. At variance, atypical (a)PKCs, such as ζ and ι/λ isoforms, are activated neither by DAG or Ca^2+^ [4,5], and are reported to be controlled by sphingosine 1-phosphate [6], phosphatidic acid and PS [7,8]. However, both the structure and mechanism regulating the activity of PKC family members have been extensively described elsewhere and go beyond the scope of this review [9].

Among the mechanisms involved, PKCs can retain an autoinhibited configuration through the binding of a regulatory pseudo-substrate, which prevents the phosphorylation of the actual substrate by the catalytic C-terminal domain [10,11]. The binding of a second messenger, e.g., DG and Ca^2+^, to their specific PKC subunits induces an open effective conformation, which releases the pseudo-substrate from the active site and provides the phosphorylation of the specific substrate. PKC signaling is strongly impaired by the dephosphorylation of the open form that is carried out by the leucine rich repeat protein phosphatase (PHLPP) and phosphatase 2A (PP2A), both responsible for addressing the kinase to the proteasome-mediated degradation. This event is biased by the binding of Heat Shock Protein 70 that allows the re-phosphorylation of PKC, proving that the balance between the phosphorylated and dephosphorylated forms is crucial for sustaining the kinase signaling within the cell. Of note, the PKC isotypes mainly involved in tumor pathogenesis reveal a compromised mechanism of auto-inhibition related to an impaired regulation by PHLPP [11]. 

For decades, PKC has been considered an oncoprotein due to its activation by phorbol esters [12], followed by the chronic down-regulation of enzyme levels [13]. Indeed, the finding that PKC isoforms act as cellular receptors for phorbol esters has also provided a possible early explanation of their own T cell stimulating capability [14]. The different isoenzymes can be identified by their distinctive regulatory region, while the catalytic domain, resembling that of other protein kinases (e.g., PKB, also known as AKT), is shared between all the family members, and includes consensus sequences for Ser/Thr residue phosphorylation similar to those observed in PKA. These homologies suggest that several PKC functions may be likely achieved by similar kinases and represent the major obstacle to the development of effective specific inhibitors, leading to off-target effects. Nevertheless, the strong involvement of many PKC isotypes in cancer development and progression has engaged many researchers to target this family of enzymes to uncover novel therapeutic approaches [15]. In this regard, a relevant common feature of tumor cells is the expression of PKC transcriptional variants, leading to either physical alteration or functional loss.

Thus, a comprehensive representation of the pathways and partners involving PKC variants may be beneficial for addressing new therapeutic strategies, to be employed either alone or in combination, and for developing more feasible molecules able to specifically target distinctive PKC isoforms. Not less critical would be a better definition of the networks communicating with PKCs and the interactions with the components of the tumor microenvironment (TME), including immune cells. Indeed, a broader view that considers the impact on the TME features of different PKC variants, either expressed by the tumor or immune cells, would help focus on specific targets and guide future efforts.

## 2. Levels of PKC and Mutations Sustaining Cancer

The critical role of PKC dysregulation in tumor promotion has been extensively described elsewhere [16]. Based on numerous research studies, it is plausible to reason that this family of kinases can also display a tumor-suppressive role by phosphorylating several downstream substrates, such as K-Ras [17]. Of relevance is the debate on a dual role of PKC as either “good” or “bad” that concerns specific isoenzymes [18], such as PKCι [19,20], PKCε [21], PKCδ [22] and PKCζ [23]. Furthermore, PKC genes are frequently mutated, especially in human cancers, with 20–25% of the mutations detected in melanoma, colorectal cancer and lung squamous cell carcinoma, whereas less than 5% are found in glioblastoma, ovarian and breast cancer [24]. A comprehensive study by Antal et al. on different tumor types has summarized a multitude of mutations (i.e., 554) affecting several members of PKC (i.e., α, β, γ, δ, ε, η, θ, ζ, ι) and occurring in both conserved and non-conserved regions [25]. Most of them are associated with a complete functional loss and affect both the regulatory and catalytic protein domains, without distinction, interfering with their own phosphorylation and catalytic potential. The most relevant and experimentally validated mutations, which we are going to discuss, are summarized in Table 1.

The first characterized mutation is the D294G, located in the C2 domain of the PKCα isoform [26]. This alteration has been reported to cause kinase functional loss and the inhibition of F-actin accumulation, thus interfering with the organization of cytoskeletal filaments at the cell–cell junctions. Another described mutation is the D463H, affecting the highly conserved Asp residue, fundamental for the kinase activity and leading to a different distribution and a reduced lifetime of the protein, likely favoring its phosphorylation. In the rare chordoid glioma, only one allele has been reported to carry this mutation, whereas the wild-type gene encodes a protein that is still able to work [29]. However, it cannot be ruled out that the inactive form encoded by the mutated allele might still interact with different partners, modifying the interactive pathways with consequences impairing the therapeutic approach of this tumor.

Selected alterations that can lead to gain-of-function are more frequently detected in Alzheimer’s disease, as in the case of PKCα M489V mutation [31]. Differently, these variants are rarely detected in cancer settings and include the SLC44A1-PKCα fusion protein, originated by the rearrangement between chromosomes 9 and 17, which generates a constitutive oncogenic and functional PKCα [32]. Other gene fusions with oncogenic effects have been found in benign fibrous histiocytomas, leading to altered PKCα, PKCβ and PKCδ isoforms interacting with membrane-associated proteins, including Podoplanin, CD63 and LAMTOR1 [34].

Numerous investigations agree with a tumor suppressive role for PKCα, which is able to inhibit the proliferation of colorectal cancer cells, and accordingly detected at lower levels in 60% of colorectal cancers [58], while a direct correlation has been reported between low PKCα and the development of intestinal tumors in PKCα^−/−^ mice [59]. PKCα activation has been described to impair the cyclin D1 translation mediated by 4E-BP1, specifically through a PI3K/AKT-independent mechanism, rather regulated by PP2A. Also, in colon cancer, PKCα has been shown to down-regulate the expression of β-catenin by inhibiting the Wnt/β-catenin pathway and, consequently, the expression of the downstream targets, such as c-Myc [18]. Further evidence of PKCα being a tumor suppressor has been provided in mice, where PKCα knockout contributes to the development of lung cancer, by upregulating K-Ras through a reduced activation of p38 Mitogen-activated protein kinase (MAPK), and a subsequent increase in Transforming Growth Factor (TGF) β1 [60].

However, this tumor suppressor competence appears to be dependent on the tumor context. Indeed, in other types of tumors, including gastric carcinoma [61], bladder [62], endometrial [63] and breast cancer [64], PKCα is clearly overexpressed, and plays different functional roles related to the distinctive substrates. Targeting PKCα is particularly suitable for the treatment of epithelial cancers, where the PDGFR-PKCα-FRA1 axis is mechanistically involved [65]. In breast cancer cells, the increase in PKCα is associated with the switch to a hormone-independent phenotype [66,67] mediated by Notch-4 [68], also representing an indicator of hormone therapy failure [69,70], and by ErbB2 up-regulation, which determines a constitutive activation of the kinase, promoting cell invasion [71]. An enhanced PKCα has been shown to induce epithelial-to-mesenchymal transition (EMT) and improved cell motility in association with alterations of E-cadherin/β-catenin expression and increase in MMP-2/MMP-9 [72]. Accordingly, these features are reverted by RNA silencing with siRNA against PKCα [65]. PKCα also displays a role in other crucial changes of cancer cells, as observed in in vitro glioma models [73], where it promotes the cell proliferation and survival in response to anticancer treatments, via activation of the NF-κB pathway [74]. Of note, the overall PKCα reduction induced by RNA interference rather than the inhibition of its catalytic activity has been implicated in restraining glioma cell growth [75].

Further evidence of the ambiguous involvement of PKC isoforms in cancer is provided by PKCβ. The PKCβII variants, together with PKCβI, represent spliced isoforms highly detected in cancer [35], but many aspects of their contribution are still poorly understood. This is due to their diverse functions, partly dependent on the different C-terminal domains conferring specific maturation processing and different cellular localization. In addition, the maintenance of the correct sequence in the C-terminal subunit of the protein is essential to the phosphorylation operated by other kinases, but the P616A and P619A mutations of PKCβII can abolish its maturation.

The overexpression of PKCβII has been associated with the early stages of tumor development [76], found in 18% of primary adenocarcinomas, and reported as a poor survival factor [77]. In transgenic mice, the up-regulation of PKCβII caused the hypertrophy of epithelial colon cells while increasing their sensitivity to the effect of carcinogens [78]. Overexpression of PKCβII has been reported to depend on either the repression of TGFβ signaling, high Cyclooxygenase-2 (COX-2) levels [79], K-Ras-mediated MEK/ERK cascade activation [80] or pathways involving other PKCs, like Ras/MEK/PKCι/Rac1 [81].

In addition, during proliferation, cancer cells can deregulate specific genes and accumulate altered PKC variants, either as the consequence of selected mutations or prolonged treatment with anticancer agents. These genetic modifications commonly translate into a loss-of-function, resulting in the generation of inactive enzymes.

The ambiguity of the functional role of PKC in cancer settings is further driven by studies regarding certain mutated isoforms, which seem rather to attribute a protective function to the canonical form [25]. A heterozygous mutation affecting the C2 domain, associated with functional loss, has also been detected in PKCβ. Of note, the induction of an exclusive wild-type PKCβII allele has been associated with a reduced anchoring ability and a decline in cell transformation and infiltration features. Since most detected mutations are heterozygous in cancer cells, the attempt to restore the endogenous PKCβ mutated gene (A509T) by using the CRISPR/Cas9 approach has led to an increase in terms of both expression levels and the basal activity of PKCβ [25]. The correction of the genetic defects has been associated with reduced cell viability and decreased ability to grow in a suspension medium. Instead, the knockout of the mutated PKCβ gene demonstrated that the presence of only one correct allele is sufficient and responsible for greater anchorage-independent cell proliferation compared with the presence of two wild-type alleles. In conclusion, hemizygous PKCβ confers lower growth potential than the co-expression of A509T mutation, proposing the former condition as dominant-negative. These findings suggest that the restoration of full PKCβ activity in mutated cancer cells could still preserve suppressive effects, debunking its oncogenic role. In synthesis, an oncogenic mutation can be favored when the functional loss of PKC activity occurs.

An extensive analysis of the whole genome and RNA-sequencing technologies have revealed a high rate of PKCβ mutagenesis in about 33% of patients with Adult T Cell Leukemia. In particular, a D427N mutation, which translates into a constitutively active PKC open conformation, is able to increase NF-κB signaling [42].

Other gain-of-function mutations have been reported for PKCγ, albeit many mutations are associated with an inactivated form of the enzyme and affect predominantly conserved motifs [17], while others, mainly occurring in colon and breast cancer have opposite effects [82,83]. In the HCT-116 colon cancer cell line, the knockdown of mutated PKCγ has been reported to decrease cell proliferation [84,85], while aberrant PKCγ expression increases cell migration [86]. In addition, the presence of a mutant form of PKCγ, not properly phosphorylated at Thr514, alters its own de novo synthesis leading to its down-regulation [82]. Of note, the M501I mutation, identified in lung cancer settings [47], has been shown to change the selectivity for the phosphorylation target in favor of a Thr, leading to the recognition of different substrates, thus deviating the kinase towards different networks [48]. From this point of view, considering the pivotal role of phospho-Thr-mediated signaling in cancer, the control of mutant PKCγ isoforms can potentially affect tumor development and metastatization [87].

About PKCδ, a loss-of-function mutation identified in gastric cancer and affecting the catalytic domain has been addressed by an interesting study by Guo et al. [49]. In this setting, the dysfunction is related to changes in the DFG and APE motifs inactivating the catalysis. However, mutations in the hinge region or in the Thr residue have been reported to completely inhibit PKCδ, preventing cleavage by caspase 3. These altered isoforms differently modulate their downstream targets, such as p53, leading to a decline in their transcription. As PKCδ is involved in the cell-to-cell interactions, these mutations hinder the generation of the tight junction complexes, altogether increasing the invasiveness of cancer cells.

Other mutations with a loss-of-function in the PKCδ gene were identified in patients affected by juvenile systemic lupus erythematosus (JSLE) patients [88], that frequently develop B cell lymphomas. The impact of PKCδ defects on its role as an immune response homeostatic regulator will be discussed below.

At the time of writing this review, no further evidence of loss-of-function mutations in PKCδ is available. Most of the studies focus on the extent of expression of this isoform, leading to very conflicting results likely attributable to the differences between the considered cell types. An enrichment in terms of PKCδ has been reported in prostate and breast cancers [89]. In the former, PKCδ promotes invasiveness with the up-regulation of PCPH/ENTPD5 leading to collagen secretion [90]. In a xenograft model of prostate cancer, PKCδ has been shown to sustain tumor growth by increasing HIF-1α-mediated angiogenesis with the involvement of the NADPH coenzyme and the production of ROS [91]. Breast cancer has been assumed to be a preferential specific context in which PKC can display an oncogenic role. In particular, high levels of PKCδ promoted tumorigenesis in both the Erb2^+^ and ER^+^ phenotypes, with the latter also correlating with poor survival [92]. In mice, PKCδ up-regulation improves resistance to apoptosis in stress conditions, such as serum deprivation or doxorubicin exposure [93]. However, in breast cancer, the effects of PKCδ are somewhat controversial. Indeed, while in BT-549 cells, the enzyme overexpression reduces the migration ability in MCF-7 cells, the decline in PKC has been reported to favor the cell migratory capacity more than its up-regulation, followed by the secretion of MMP-9. So, the inhibitory effect of PKCδ on cell growth has been reported with different consequences based on the type of cells. PKCδ can drive cell cycle arrest in G1, mediated by the induction of p21cip1 and Rb dephosphorylation [94], although a block in the S phase has also been observed as a consequence of PKCδ overexpression [95]. Several findings underline the PKCδ involvement in apoptosis as a response to chemotherapy [96], and a relocation of the kinase into the nucleus has been observed after exposure to genotoxic drugs [97]. In androgen-dependent prostate carcinoma, apoptosis is rather allosterically activated through either the p38 MAPK/RhoA/ROCK/p21Cip1 [98,99], or the TNF-α/TRAIL/JNK/p38 MAPK caspase 8 networks [100,101,102]. It is then clear that PKCδ is engaged in so many and complex different processes (e.g., regulation of cell cycle, motility and metastasis diffusion) that it might hardly represent a feasible tumor-associated therapeutic target.

Concerning PKCε, many experimental observations link the pro-oncogenic functions of this isoform to aggressive tumor phenotypes, making PKCε a good target for anticancer applications. In particular, the kinase has been reported as engaged in the Ras-Raf-1 signaling [103,104] and in the autocrine TGFβ secretion loop [105]. PKCε is increased in about 75% of patients affected by invasive ductal breast cancer and correlates with the severity of the disease in association with ErbB2^+^/Her2^+^ and ER^-^ and PR^-^ phenotypes [106]. Accordingly, at the cellular level, the PKCε isoform has been shown to inhibit tumor cell apoptosis by blocking the transfer of BAX to the mitochondria [107]. Even greater seems to be the correlation of PKCε with the growth of primary epithelial non-small-cell lung cancer (NSCLC, >90%), in which it is activated in a p21/Cip1-dependent way [108]. Again, PKCε levels are elevated in prostate cancer as compared with benign prostatic epithelia and are associated with higher aggressiveness [109]. Indeed, in human prostate cancer, PKCε participates in survival mechanisms, modulating caspases and Bcl-2 [100,110,111] while modulating invasiveness by activating RhoA and/or RhoC [106,112]. Increased PKCε expression levels and activation have been reported in prostate tumors, where the isoform regulates ERK, AKT and mTOR pathways [18,113,114], the anti-apoptotic TNF-α-dependent NF-κB signaling and the expression of survival and invasive factors (e.g., COX-2, MMP-9, cyclin D1, VEGF, IL-6), through STAT 3 [115]. Finally, the interaction between PKC and RACK1 has been shown to interfere with integrin β, mediating relevant changes in terms of focal adhesion and cell motility also through ERK [116,117]. Indeed, PKCε has been reported to support the assembly of the adhesion matrix containing actin filaments and β1-integrins, the latter further linking PKCε to the AKT survival pathway in recurrent prostate cancer cells [114]. 

PKCζ and PKCι (PKCλ) represent atypical PKC, i.e., lacking a C2 domain and endowed with only an unresponsive C1 domain [118,119,120]. PKCζ acts predominantly as a suppressor by inducing apoptosis in different tumors [121]. In addition, PKCζ limits IL-6 secretion through the inhibition of the Ras cascade [122]. Its deletion in PTEN-knockout mice has been reported to promote prostate carcinoma, also in correlation with the c-Myc increase [123]. Only a mutated form of PKCζ, constitutively activated, has been described in a mouse transgenic model of prostate adenocarcinoma [51]. Since PKCι works as an oncogene, it is often overexpressed in cancer [124,125], sometimes depending on gene amplification [126,127], due to the association of the *PRKCI* gene to the 3q amplicon [54]. The tumorigenic effects of PKCι have been described as mediated by the Rac1-PAK-MEK-ERK network [80], but alternative ways are described, including the Rho-GEF-Ect2 pathway, involved in the formation of the PKCι-Par6 complex [128]. Other links are reported to the NF-κB signaling [129,130].

In recent years, more attention has been addressed to the PKCι isoform. This is significantly overexpressed in pancreatic ductal adenocarcinoma, one of the more lethal tumors, as a marker associated with disease progression and poor prognosis [131,132]. PKCι acts on the PI3K/AKT and Wnt/β-catenin pathways, opposing apoptosis. Of relevance, the RNA interference has shown to reduce proliferation, migration and invasive behaviors of cancer cells, advocating PKCι as a feasible target for therapy. Novel investigations indicate that PKCι activities, in this context, could depend more on the interaction with other protein partners, rather than on its kinase function. Indeed, a dibasic motif has been identified in some substrates, such as lethal giant larvae 2 and myosin X (but absent in Par3), containing two essential residues: Arg471, involved in the binding of lethal giant larvae 2, and Arg474, involved in the binding of myosin X. A mutation that can frequently occur in this motif was previously misnamed R471C and was recently renamed as R480C [55]. This region of PKCι participates in the cell polarization during epithelial morphogenesis; however, the mutation modifies the recruitment of the corrected target substrates, altering the process [56]. Of relevance, the 3q26-29 amplicon including the *PRKCI* gene is reported as pathogenic by https://www.ncbi.nlm.nih.gov/clinvar/ (accessed on 15 July 2023) (GRCh38/hg38 3q26.1-29(chr3:166137209-198125115)x3) and has been suggested as a marker of small-cell lung cancer (SCLC) [54].

Collectively, these observations suggest that the occurrence of mutations increases the complexity of interactions and biological activities regulated by PKC isoenzymes, thus contributing to restraining the development of selective PKC modulators.

## 3. The Challenging Task of Targeting PKC for Cancer Therapy

PKC isoenzymes are found at the intersection of multiple signaling pathways in many physiological and pathological conditions. The frequent dysregulation of PKC isoforms observed in many cancers, and the evidence that PKC plays an important role in cell transformation, differentiation, migration, and tumor progression, has generated considerable interest in the development of specific PKC modulators for cancer treatment [10,15,133].

Several strategies have been employed, aiming to discover the selective inhibitors of PKC, mostly competing with ATP to the C3 region within the catalytic domain of the kinase, or binding either to the C4 (substrate competitive PKC inhibitors) or the C1 domain (DAG competitive molecules) in the regulatory region. The PKC isoform-specific antisense oligonucleotides have also been described.

Many PKC modulators are presently at various stages of development in the clinical setting; however, most of these trials have failed to show a significant clinical benefit [10,15,133], which strongly limits the ability to develop cancer treatment strategies by targeting PKC alone. Below is a brief characterization of the main PKC inhibitors employed in both preclinical and clinical settings in different types of tumors.

### 3.1. Staurosporine

The best-studied ATP competitive PKC inhibitor is staurosporine, a molecule isolated from *Streptomyces staurosporeus* bacterium, with an IC_50_ value of 2.7 nM [134]. This compound binds to the catalytic domain of all PKC isoforms and exhibits unspecific inhibitory activity against numerous other Ser/Thr as well as tyrosine kinases. Indeed, this lack of selectivity restricts its applicability in clinical studies, nonetheless, several staurosporine analogs, such as 7-hydroxystaurosporine (UCN-01), 4’-N-benzoylstaurosporine (midostaurin), enzastaurin, AEB071 (also known as sotrastaurin), have been developed and used in clinical trials either as single agents or in combined therapeutic approaches in patients with different types of cancer [9].

### 3.2. UCN-01

UCN-01 is a potent inhibitor of multiple protein kinases, including PKC and cyclin-dependent kinases (CDKs). UCN-01 blocks PKC activity (IC_50_ 4.1 nM) by binding to its catalytic domain [135] with significantly higher affinity for conventional (c) PKC than for novel (n) PKC and atypical (a) PKC [136], disturbing the transition from the G1 to S phase, thus preventing cancer cell growth [137].

Preclinical studies have observed a synergistic activity of UCN-01 when combined with several cytotoxic agents [138]. Based on these results, several Phase I studies have been carried out with UCN-01 either as a monotherapy or in combination with cytotoxic chemotherapeutic agents in patients with solid tumors, leukemia and lymphomas [139,140,141]. 

UCN-01 can be safely administered as an initial 72 h continuous intravenous infusion, followed by monthly doses given as a 36 h administration, showing good tolerability. In addition, this agent has a very small volume of distribution, low systemic clearance and a prolonged elimination half-life [141]. UCN-01 was tested in Phase I trials in combination with either cisplatin [140] or 5-fluorouracil [139] or topotecan [142] or carboplatin [143], or irinotecan [144,145] in advanced solid tumors. Other phase I studies combining UCN-01 with prednisone in patients with refractory solid tumors and lymphomas [146], and with perifosine in patients with relapsed and refractory acute leukemias and high-risk myelodysplastic syndrome [147] have been carried out, while a combined approach with fludarabine monophosphate has been investigated in patients with chronic lymphocytic leukemia [148]. Also, a Phase II clinical trial with irinotecan has been conducted in patients with metastatic triple negative breast cancer who had previously been treated with anthracyclines and taxanes [145]. Unfortunately, most of these investigations failed to show clinical benefits, which has discouraged further clinical trials with UCN-01.

### 3.3. Midostaurin

The staurosporine semi-synthetic analogue midostaurin (4’-N-benzoylstaurosporine also known as PKC412; CGP 41251) is an ATP-competitive inhibitor, isolated from *Streptomyces Staurosporeus* with a higher specificity for PKC (PKCα, IC_50_ 22 nM; PKCβ, IC_50_ 30 nM) compared to its parental compound. Initially developed as a PKC inhibitor analogous to staurosporine, midostaurin inhibits several other protein kinases, although with a lower inhibitory activity on PKC compared with staurosporine. Accordingly, its therapeutic effects are mainly attributed to the inhibition of tyrosine kinases [149]. In promising preclinical studies, midostaurin has shown anti-tumor activity both in vitro and in vivo, and the capacity to reverse P-glycoprotein (Pgp)-mediated multi drug resistance (MDR) [150,151,152,153]. Of note, it has also displayed potent anticancer effects against rituximab resistant Burkitt’s lymphoma cells by decreasing PKC phosphorylation and enhancing pro-apoptotic activity [154].

The compound was then tested in 56 clinical trials in patients with various tumor types, either as a monotherapy or in combination with other drugs, showing some survival benefit in patients with selected cancers ([155], https://clinicaltrials.gov/ct2/results?term=midostaurin, accessed on 13 June 2023). However, although its good tolerability was demonstrated in a Phase I trial, with the most common toxicities including nausea, vomiting, diarrhea and fatigue, in a Phase II study conducted in patients with malignant melanoma, midostaurin failed to demonstrate a statistically significant clinical activity [156]. 

In patients with AML, FMS-like tyrosine kinase 3 (FLT3) mutations with internal tandem duplication (ITD) are associated with high leukemic burden and poor prognosis [157]. Of relevance, the addition of midostaurin to standard chemotherapy has significantly prolonged median overall and event-free survival in mutant FLT3-positive AML patients in a Phase III study [158]. Nevertheless, the direct inhibition of tyrosine kinase by midostaurin resulted in G1 arrest and apoptosis in FLT3-mutant leukemia cells [149]. Since April 2017, midostaurin has been approved by the Food and Drug Administration (FDA) for the treatment of patients with newly diagnosed FLT3-mutated AML or patients with systemic mastocytosis with associated blood neoplasm or mast cell leukemia. More recently, a high response rate and good tolerability with no evidence of increased toxicity have been shown with the use of the anti-CD33 immunoconjugate gemtuzumab-ozogamicin plus midostaurin added to standard intensive chemotherapeutic regimens in patients with newly diagnosed FLT3-mutated/CD33^+^ AML [159]. However, a larger cohort of patients with longer follow-up is needed to validate these results. 

Of note, the oral administration of midostaurin has displayed significant clinical improvements also in patients with advanced systemic mastocytosis in a Phase II study [160].

### 3.4. Sotrastaurin

Sotrastaurin {AEB071; 3-(1H-indol-3-yl)-4-[2-(4-methylpiperazin-1-yl) quinazolin-4-yl] pyrrole-2,5-dione} is a first-generation oral potent ATP competitive inhibitor, that blocks the catalytic activity of both the classical (α, IC_50_ 0.95 nM; β, IC50 0.64 nM) and novel (δ, ε, η,θ, IC_50_ ranging from 0.22 to 3.2 nM) isoforms of PKC [161]. In preclinical studies, it has been shown to considerably reduce the viability of uveal melanoma cells harboring GNAQ/GNA11 mutations by inhibiting PKC isoforms, specifically through the PKC/NF-κB and PKC/ERK1/2 pathways [162,163]. More than 90% of patients with metastatic uveal melanoma (MUM) have mutations in the *GNAQ* and *GNA11* genes, which encode the alpha subunits of the G-protein-coupled receptor (GPCR) [164]. The phospholipase C/PKC signaling pathway is an important downstream player of the constitutively active G protein alpha subunits (GNAQ or GNA11) [165]. Therefore, PKC inhibitors may be considered as a valuable option for treating MUM. A recent Phase I dose-escalation study, involving patients with MUM treated with sotrastaurin, has exhibited favorable tolerability and moderate clinical activity, although with a low objective response rate (3%) [166].

Sotrastaurin has also been tested in a Phase Ib trial in patients with MUM in combination with the PI3Kα inhibitor alpelisib to achieve a sustained suppression of MAPK signaling. Dual therapy was found to be safe, but there was no evidence of clinical efficacy [167].

Darovasertib (LXS196) is a new second generation oral selective PKC inhibitor (PKCα, IC_50_ 1.9 nM; PKCθ, IC_50_ 1.9 nM) (https://pubchem.ncbi.nlm.nih.gov/docs/compounds, accessed on 17 July 2023) that demonstrates enhanced pharmacological activity against UM cell lines containing mutant *GNAQ* or *GNA11* [168] when compared to sotrastaurin. Currently, several clinical trials (https://clinicaltrials.gov.,identifier: NCT03947385, accessed on 17 July 2023) are underway to evaluate the safety and anti-tumor activity of darovasertib in patients with solid tumors harboring GNAQ/11 mutations, including MUM, cutaneous malignant melanoma and colorectal cancer. 

In a first-in-human Phase I study, darovasertib was well tolerated, with hypotension being the most common dose-limiting toxicity. The compound also proved to encourage clinical activity as a single agent in MUM patients [168].

### 3.5. Enzastaurin

Enzastaurin (LY317615) is an ATP-competitive, selective inhibitor of PKCβ (PKCβ, IC_50_ 6 nM; PKCα, IC_50_ 39 nM; PKCγ, IC_50_ 83 nM; PKCε, IC_50_ 10 nM) belonging to the class of acyclic bisindolylmaleimides [169], that also exhibits antiproliferative and proapoptotic effects by targeting AKT and GSK3β kinases [169].

Enzastaurin has been investigated in various clinical trials for the treatment of different types of cancer. At the time of writing this review, 51 clinical trials with enzastaurin alone or combined with standard treatments, were listed on https://clinicaltrials.gov (accessed on 17 July 2023). Although enzastaurin has shown promise in both preclinical studies and distinctive patient populations, demonstrating a favorable toxicity profile in many Phase I studies [170], further research is required to determine its actual efficacy in the clinical setting. In fact, no clear benefit has been observed in several clinical trials evaluating the employment of enzastaurin alone or in combination with other anticancer agents, such as erlotinib or pemetrexed, in patients with advanced NSCLC [171,172], bevacizumab or temozolomide in patients with recurrent malignant gliomas [173,174], docetaxel/prednisone in patients with castration-resistant metastatic prostate cancer [175], and with paclitaxel and carboplatin in patients with advanced ovarian cancer [176]. Unfortunately, the lack of significant clinical improvement after treatment with enzastaurin has also been observed in previously treated patients with multiple myeloma [177], brain metastases [178], epithelial ovarian or primary peritoneal carcinoma [179], refractory mantle cell lymphoma [180], metastatic breast cancer [181] and refractory advanced cutaneous T cell lymphoma [182]. 

According to the current “precision medicine approach”, a new biomarker in diffuse large B-cell lymphoma (DLBCL), called DGM1 (De novo Genomic Marker 1, a germline polymorphism) located on chromosome 8, has been correlated with response to enzastaurin. These data suggest that adding enzastaurin to the standard-of-care R-CHOP protocol (cyclophosphamide, doxorubicin hydrochloride, vincristine sulfate, rituximab and prednisone) may improve the outcomes in high-risk DGM1 + DLCBL patients [183].

### 3.6. Bryostatin

An additional class of PKC modulators with anti-cancer activity are the bryostatins, which are macrocyclic lactones derived from the marine invertebrate bryozoan *Bugula neritina*. Bryostatin-1 (binding affinity for PKC Ki, 1.35 nM, https://pubchem.ncbi.nlm.nih.gov/compound/, accessed on 17 July 2023) competes with cancer-promoting PKC ligands such as DAG and phorbol 12-myristate 13-acetate (PMA) to bind to the C1 regulatory domain of PKC-promoting either activation (short-term exposure to the drug) or inhibition (long-term exposure to the drug) of PKC [184]. The ability of PMA to induce a broad range of effects, including maturation/differentiation/apoptosis in hematopoietic cell lines at very low concentrations, has stimulated investigators to administer the phorbol ester to patients with myeloid leukemias. PMA has so far only been used in clinical studies aiming to establishing its dose tolerance [185], with bryostatin-1 being the most widely used compound in clinical trials.

Clinical trials using Bryostatin-1 as monotherapy or combined with other anticancer agents have failed to demonstrate significant clinical benefits in a variety of cancers, including melanoma [186], renal cell carcinoma [187], colorectal cancer [188], non-Hodgkin’s lymphoma (NHL) [189], relapsed multiple myeloma [190], sarcoma, head and neck cancer [191], cervical cancer [192], ovarian cancer [193], pancreatic cancer [194] and NSCLC [195]. Of relevance, the combination of bryostatin-1 and cisplatin has been associated with increased toxicity (mainly severe myalgias) in patients with ovarian cancer pre-treated with platinum [193]. Also, in a clinical study combining bryostatin-1 with paclitaxel in patients with NSCLC, increased myalgia has been reported [195]. On the other hand, a Phase II trial of sequential treatment with paclitaxel and bryostatin-1 in patients with untreated advanced gastric or gastroesophageal junction cancer, displayed a higher response rate than paclitaxel alone [196]. Unfortunately, a subsequent clinical investigation using the same treatment schedule in patients with advanced esophageal cancer was stopped early due to excessive toxicity, despite potential good anti-cancer activity [197]. According to this potential benefit, a Phase II trial, combining bryostatin-1 and vincristine, has demonstrated efficacy in a subset of patients (with an overall response rate of 31%) with aggressive B-cell NHL who had relapsed after autologous stem cell transplantation [198].

### 3.7. Antisense Oligonucleotides

Another approach to targeting PKC involves the use of antisense oligonucleotides, which can selectively reduce the expression levels of PKC isoforms implicated in cancer cell proliferation, apoptosis and distinctive signaling pathways. The anti-tumor activity of antisense oligonucleotides, as well as their safety and tolerability, have been confirmed in many studies [199,200].

Aprinocarsen (ISIS-3521/LY900003) is a 20-base phosphorothioate antisense oligonucleotide designed to specifically bind to the 3′-untranslated region of human PKCα mRNA [201]. The hybrid complex is then cleaved by RNase H, leading to the inhibition of PKCα expression (IC50 value of 50–100 nM for PKC-α mRNA reduction). Aprinocarsen has been used in Phase II and III clinical trials in patients with recurrent high-grade astrocytoma [202], advanced NSCLC [203], advanced ovarian cancer [204], hormone-refractory prostate cancer [205], breast cancer, [206], colorectal cancer [207] and previously treated low-grade NHL [208]. The drug was well tolerated, with fatigue and thrombocytopenia reported as the most common side effects [200].

However, since most of these trials failed to show an improvement in terms of either survival or other clinical benefits when used alone or in combination with anticancer drugs [202,203,204,205,206,207,208], the use of aprinocarsen in clinical trials was discontinued in 2005 [209].

Therefore, it is important to emphasize that the development of new PKC inhibitors with greater isoenzyme specificity and a better understanding of the involved signaling pathways may lead to more effective therapeutic approaches.

Most relevant clinical studies involving the use of PKC inhibitors in cancer settings are summarized in Table 2.

## 4. PKC Isoforms in Anticancer Immune Responses: The Bad and the Good Guys in the Family

### 4.1. Impact of PKC on T Cell Responses and B Cell Development

By using either knock-down animal models, PKC inhibitors, or PKC-defective T cell lines, many either redundant or unique biological roles have been established for the different PKC isozymes, impacting different pathways required for a proficient T cell response, and extensively reviewed by Pfeifhofer-Obermair et al., back in 2012 [210]. Currently, renewed attention must undeniably be paid to the distinctive PKC isoforms involved in the different aspects of the immune response in light of their potential impact on immune checkpoint blockade (ICB)-induced immune re-invigoration [211]. 

T cells have been shown to display up to eight distinctive types of PKC members [210], which hinders a detailed categorization of the individual roles. 

#### 4.1.1. PKCθ

Among PKC isoforms, PKCθ is critical for the activation of NF-κB, AP-1 and NFAT transcription factors, a prerequisite for mature T cell stimulation and survival [212]. In particular, PKCθ promotes CARMA1 serine phosphorylation, required for its ability to stimulate NF-κB [213] through the recruitment of the Bcl10-MALT1 complex, which in turn leads to the degradation of IκB, resulting in NF- κB activation and nuclear import [214].

Crucial to an efficient T cell stimulation is the recruitment of PKCθ to a small central area of the immunologic synapse (IS), the supramolecular activation complex (SMAC), where it engages with several proteins which are instrumental for the generation of signaling complexes, including the SRC family tyrosine kinase LCK, which contributes to the phosphorylation of PKCθ mainly at Tyr90 in the regulatory domain [215], the lymphocyte function-associated antigen 1 (LFA-1) [216], and the IL-2-inducible T cell kinase (ITK) [217], while negatively regulating the type E3 ubiquitin ligase Cbl-b [218], which restrains the PI3K activity [219]. Of relevance, PKCθ facilitates the engagement of LFA-1 to its own ligand intercellular adhesion molecule 1 (ICAM-1), expressed on activated APCs [216], then facilitating the generation of a more stable IS. Moreover, the increased adhesion potential to the endothelium delivered by the LFA-1 and LFA1-ICAM engagement with cancer cells may serve as a mechanism entrapping activated CD8^+^ T cells in the tumor site [220], thus potentially contributing to the generation of a more inflamed TME.

The enrolment of these molecules is supported by the engagement of PKCθ with the cytoplasmatic tail of the co-stimulatory molecule CD28 [221], required for its own translocation to the membrane [222], followed by interaction with AKT kinase, which preserves its critical role as IS rheostat [223]. In T cells, in the absence of CD28, PKCθ is diffused throughout the synapse, indicating that CD28 is specifically required for PKCθ localization to the central (c)-SMAC region and the establishment of a mature IS [222]. This is of critical importance since CD28 represents the main down-stream target of PD-1-mediated inhibition through the SH2 domain-containing protein tyrosine phosphatase 2 (SHP-2) [224] and is required for T cell re-invigoration following PD-1 blockade [225]. A nonconventional PI3K- and Vav-associated signaling pathway has also been reported to facilitate a specific PKCθ membrane recruitment and activation by anti-CD3/CD28 stimulation in T lymphocytes [226]. Of note, PKCθ represents, along with CD3ζ-associated protein of 70 kD (Zap70), another direct target of the inhibition to the TCR downstream signaling resulting from PD-1 engagement in T cells [227], and leading to impaired activation, proliferation, cytokine production and metabolic reprogramming [228]. This identifies PKCθ as a sensible therapeutic target to increase the effect of ICB and overcome therapeutic resistance in selected patients.

Among the mechanisms operating upstream PKCθ, MAP4K3 GCK-like kinase (GLK) is responsible for kinase Thr538 phosphorylation [229], a prerequisite for the activation of IL-2 promoter by NF-κB and NFAT in mature T cells [230], and for retaining PKCθ at the IS. A further level of control downstream of TCR is represented by the DG kinases (DGK) α and ζ, which facilitate DAG consumption, thus impairing the availability required for PKCθ activation [231]. 

The assessment of polyfunctionality, as defined by the contemporary/sequential release of different cytokines by activated T lymphocytes [232], finely mirrors an ongoing and effective anti-tumor T cell functional activity. PKC has been shown to play a critical role in the orchestrated release of effector cytokines TNF-α, IFN-γ and IL-2, through the involvement of distinctive mechanisms [233]. Following an appropriate stimulus, while an early yet short-lived TNF-α production has been described, the release of IFN-γ has been reported as more persistent, and IL-2 production mainly depending on de novo gene transcription. Nevertheless, while PKC drives TNF-α translation without stabilizing its mRNA, the permanence of IFN-γ and IL-2 transcripts is sustained by the kinase. Since the PKC isoenzymes involved in sequential cytokine production are not specified, these observations suggest that a better comprehension of the involvement of the different PKC isoforms in the mechanisms regulating distinctive cytokine generation can contribute to improving the quality of polyfunctional anti-tumor responses. 

Nevertheless, a normal protective Th1 immune response, with an intact CD8^+^ effector memory T cell activity, has also been observed in PKCθ-deficient mice [234], which suggests a redundant control by other kinases. At variance, the absence of PKCθ has been associated with severe defects in both Th17 and Th2 responses, the latter likely associated with the PKCθ-mediated expression of the Th2 differentiation controller GATA-3 [235]. 

PKC-θ has also been involved in the molecular mechanisms regulating the rapid NK cell discrimination between healthy and malignant cells. A recent original study by Ben-Shmuel et al. [236] has established a role for PKCθ in fine-tuning the balance between stimulatory and inhibitory signals by controlling SHP-1 phosphatase, in the early stages of activation and/or inhibition of NK cell synapse. In particular, the phosphorylation of SHP-1 on the Ser591 residue by PKCθ supports a repressed SHP-1 state, while neutralization of PKCθ has been reported to preserve SHP-1 into an active conformation, reducing NK cell activation and cytotoxicity, thus promoting tumor progression in vivo. This is of relevance since SHP-1 can provide PD-1-mediated downstream dephosphorylation in the absence of SHP-2 [237], while another IC molecule, the B and tumor activity of T cells T lymphocyte attenuator (BTLA) [238], has been reported to engage SHP-1 in preference to SHP-2 [239] to impair lymphocyte functionality. 

Human T cells can proliferate to a limited extent before entering replicative senescence [240,241], characterized by a progressive telomere shortening, following each round of antigen (Ag)-induced duplications [242] that can be overcome by the enzyme telomerase, which is activated by T cell stimulation [243]. The activation of telomerase in stimulated T cells has been shown to require the PKC-dependent stimulation of the hTERT catalytic subunit expression [244], although T lymphocytes activated for different lengths of time may employ distinctive mechanisms to express hTERT. PKCθ-induced expression of hTERT is indirectly mediated by NF-Κb through an increased binding of c-Myc and Sp1 transcription factors to their sites on the hTERT promoter [245], thus providing a proliferative advantage for Ag-specific T cells. 

PKCθ expression has been previously restricted to the lymphoid lineage, while more recent investigations have defined a role in the response of macrophages to infection [246], suggesting that it also impacts myeloid features by modulating cytokine release and the potential for antigen (Ag) presentation.

Immunosuppression may partially be mediated through the recruitment and induction of FoxP3^+^ regulatory T (Treg) cells that provide the required maintenance of the balance amid tolerance to self-Ags and an effective anti-tumor response [247].

Like for conventional T cells, TCR signals are also critical for the development and activation of Treg-mediated inhibitory function. At variance with functional effector T cells, in Tregs, PKCθ is segregated from the central domain of the IS [248]. Of note, through this differential positioning within the IS, PKCθ would potentially promote the activation of effector T cell functions over Treg inhibitory activity [249]. Accordingly, Ma et al. [250] have proposed that the differentiation of induced (i)Treg cells is inhibited by the PKCθ-mediated AKT-Foxo1/3A pathway [250], while an increase in iTreg-mediated cell immunosuppression has been reported following kinase neutralization [251]. 

At variance, Gupta et al. showed a substantial reduction in the number of natural (n)CD4^+^Foxp3^+^ Treg cells in mice deficient in PKCθ [252]. The kinase has been reported to control the generation of Tregs, likely through the activation of the calcineurin/NFAT-dependent Ikaros, required for an effectual suppression [253]. Also, PKCθ appears to be required for the effective generation of immunoregulatory CD4^+^ T cells expressing IL-10 [254], thus contributing to the preservation of peripheral tolerance.

Therefore, according to these apparently contrasting results, the precise impact of PKCθ on the signaling controlling effector versus Treg phenotype development has not been completely elucidated and requires further research. However, opposing roles appear to be played by PKCθ in the generation of natural and peripherally induced Tregs.

#### 4.1.2. PKCα

Alongside PKCθ, the conventional PKCα also plays an important role in promoting an effective T cell-mediated immune response, firstly through a vigorous impact on the differentiation and expansion of immature thymocytes. PKCα is also directly involved in the activation of the PI3K/AKT pathway following TCR activation, by inducing the Ser473 phosphorylation of AKT [210], critical for the kinase full activation. AKT represents a main molecular target of the PD-1 inhibitory activity in T lymphocytes [255,256] and determines a polyfunctional phenotype in Ag-specific CD8^+^ T cells derived from the peripheral blood of melanoma patients [257]. 

However, in discriminating redundant versus individual functional activities, PKCα has been reported to play a beneficial role in T cell activation, either along with PKCθ, e.g., through the IκB/NF-κB axis, or via the engagement in distinctive pathways [258]. A fast and transient engagement to the IS in the DG-based signalosome induced by Ag recognition has also been described for PKCα, leading to the activation of Ras/ERK pathway and shedding of CD62L (L-selectin) [259], favoring the progenitor differentiation into effector memory T cells (TEM), and their migration toward peripheral tissues [260], including tumor sites. Moreover, T cells defective for PKCα fail to stimulate the TCR/NFAT/AP-1 axis, which is involved in the activation of IL-2 and IL-17A promoters [261]. The non-redundancy of roles played by the two kinases in the regulation of T cell development and activation is further advocated by the strong immunosuppressive phenotype derived by the dual PKCθ/PKCα inhibition with sotrastaurin [262].

#### 4.1.3. PKCδ

Among the other isozymes, PKCδ has been reported to control B cell growth and negatively control T cell responses [263]. Gruber et al. demonstrated an increase in terms of both proliferation and IL-2 release in T cells isolated from PKCδ-deficient mice [264], while the interaction of PKCδ with CARMA1 has been reported to impair NF-κB activation [265]. A critical function for PKCδ in supporting T cell apoptosis has also been reported, following the cytokine deficiency and engagement of Fas, leading to a caspase 3-mediated proteolytic activation [266]. Indeed, when activated, PKCδ translocates to the nucleus, where it supports T cell apoptosis via deliver of cytochrome c, PARP cleavage, histone phosphorylation and caspase 3 stimulation [267].

However, besides these negative regulatory effects, the Thr505-phosphorylated form of PKCδ has been reported to support CD8^+^ cytotoxic T cell response by co-localizing with secretory lysosomes and mediating granules’ mobilization and exocytosis in response to TCR signals [268]. More recent studies have suggested that both processes, namely microtubule-organizing center (MTOC) polarization to the IS and granule exocytosis, are controlled by PKCδ, likely through two distinctive and coordinated pathways, involving the reorganization of F-actin at the IS and at the centrosomal area, respectively [269]. Authors have suggested that the existence of two regulatory mechanisms coordinated by PKCδ can establish a fine-tuned production of regulatory checkpoint molecules, supporting a critical role for PKCδ in maintaining T cell homeostasis. Two main types of vesicles have recently been identified based on their content and the requirements of the signal for degranulation: light vesicles carrying FasL and 15 kDa granulysin, released in a PKC-dependent and Ca^2+^-independent mode and dense granules containing perforin, GRZ and 9 kDa granulysin, which require additional Ca^2+^-mediated signals [270,271]. Notably, both types of vesicles also convey several other immunomodulatory proteins including MHC class I and II, costimulatory and adhesion molecules, enzymes (i.e., CD26/DPP4), also endowed with co-stimulatory functions [272] or cytokines, indicating a wider spectrum of influences on different aspects of T cell functional activity.

Of note, by using PRKCD-defective mice, a critical role for PKCδ in limiting the autoimmune features of SLE by restraining the accumulation of autoreactive B cells has been established, thus proving human PKCδ deficiency as crucially involved in autoimmunity generation [273]. Indeed, CD4^+^ T cells from PKCδ defective mice also display distinctive defects, including impaired ERK signaling, reduced DNA methyltransferase 1 (DNMT1) expression and the increased methylation of genes implicated in the amplified immune response scenario representative of lupus [274], supporting a critical role for PKCδ as an immune gatekeeper, as suggested by Salzer et al. [273].

#### 4.1.4. PKCε

At variance with PKCδ, an anti-apoptotic role has been suggested for novel kinase PKCε through the inactivation of BAD, mediated by p90Rsk-dependent phosphorylation [275]. Numerous studies have shown a redundant role for PKCε in terms of the positive regulation of the NF-κB/NFAT/AP1 pathway [210] and in the recruitment at the center of the IS and MTOC [276], shared with PKC-θ. At variance, the regulation of T cell proliferation and the contribution to a reduced T cell sensitivity to TGFβ1, shown by Mirandola et al. [277] are reasonably not redundant. This is of critical interest, considering the role of TGFβ1 in promoting both the generation of Tregs [278] and T lymphocytes with features of tissue-residency, the latter through the induction of integrin CD103 [279,280]. A considerable fraction of tumor-infiltrating CD8^+^ T lymphocytes is represented by this tissue resident memory cell (T_RM_) population, which has emerged as one of the key tumor-reactive T cell subgroups able to exert optimal protective immunity. Accordingly, a key role for CD103^+^CD8^+^ T_RM_ has also been described in terms of response to PD-1 blockade [281]. 

T_RM_ accumulate in different human cancers, including NSCLC, and are characterized by the expression, besides CD103, also of C-type lectin CD69, which both contribute to their residency features [282]. A seminal work by Le Floc’h [283] described the involvement of the PLC/PKC signaling pathways in CD103-dependent TCR-mediated cytotoxicity. A role for PKCδ in MTOC cytolytic granule polarization and degranulation has also been described above [268]. Of note, the engagement of CD103 in tumor-specific cytotoxic T cells with its own ligand E-cadherin has been reported to increase the interaction with the target cell, likely lowering the threshold for the TCR/CD3 signal activation while supporting polarization and granule delocalization throughout the stimulation of PLC/PKC.

#### 4.1.5. PKCη

PKCη, another member of the novel PKC subfamily, is also highly expressed in T cells, endowed with several redundant functions shared with PKCθ. Notably, this isoform is recruited to the IS upon TCR stimulation, but unlike PKC θ, which is concentrated at the central region of the synapse, PKCη diffuses over the whole synapse area [284], as described for PKC θ in the absence of CD28. Since T cell differentiation correlates with the loss of co-stimulatory molecule CD28 [285], distinctive and/or cooperative roles for the two isoforms in the TCR-associated signalosome according to the differentiative status of T cells can be hypothesized. 

PKCη-deficient Treg cells have shown defective suppressive activity. Of note, PKCη associates with and recruits CTLA-4, required for contact-dependent suppression, along with the GIT2–αPIX–PAK complex. The defective activation of this complex in PKCη-deficient Tregs has been associated with a reduced depletion of the co-stimulatory molecule CD86 from APCs, promoted by CTLA-4, implicating this pathway as a potential anti-cancer immunotherapeutic target [286]. Accordingly, the reduced development of both B16–F10 melanoma and TRAMP-C1 adenocarcinoma tumors have been reported in the preclinical models of PKCη defective Tregs, in association with a less immunosuppressive TME, increased the frequency and functionality of tumor-infiltrating CD8^+^ effector T cells and an elevated expression of CD86 on intra-tumoral DCs. At variance, PKCη defective CD8^+^ T cells have not been reported to impact either tumor growth or the frequency and functionality of CD8^+^ effector T cells [287], proving that the acquisition of a more inflamed TME can be achieved by indirectly targeting intra-tumor Treg PKCη.

#### 4.1.6. PKCζ and PKCι

The PKCζ isotype has also been directly involved in the differentiation of Th2 T cells [288] and in IL-2-mediated proliferation [289], thus showing redundancy but also cooperation with PKCθ, especially in the activation of NF-κB [218,261].

**Figure 1:**
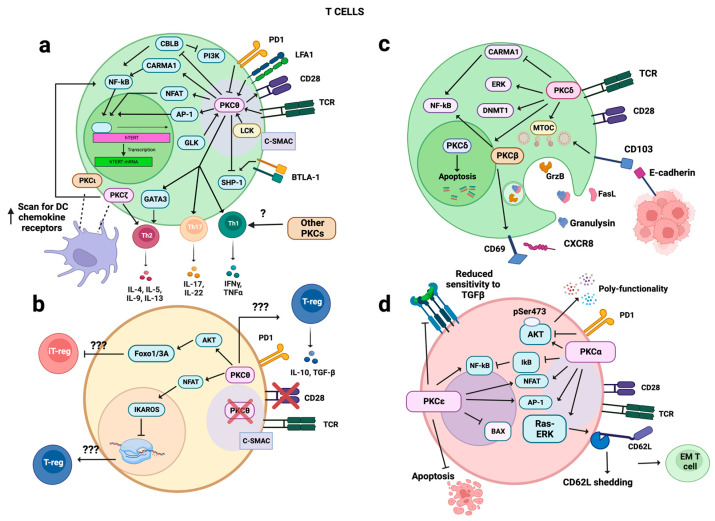
Impact of the main PKC isoforms on different aspects and inter-players of T cell functionality. (**a**) The main roles of PKCθ and interactions with inter-players involved in T cell activation; localization of PKCθ within the cSMAC in the presence of CD28; role of PKCθ in T cell differentiation; role of PKCζ and PKCι in terms of DC scan for chemokine receptors and of PKCζ in terms of Th2 cell differentiation. (**b**) Localization of PKCθ outside the cSMAC in the absence of CD28 and in Tregs; impact of PKCθ in terms of either favoring/inhibiting Treg differentiation. (**c**) Negative impact of PKCδ on T cell activation and positive influence on MTOC polarization and degranulation, positive impact of PKCβ on T cell activation. (**d**) Positive effect of PKCα on T cell activation and on TEM development via CD62L shedding; positive effect of PKCε on T cell activation and protective effects from T cell apoptosis; induction of reduced sensitivity to the effects of TGFβ. Created with https://www.biorender.com (accessed on 28 June 2023).

A redundant role for PKCζ and PKCι, both required for T cell motility and the ability to scan DCs for chemokine receptors has been suggested [290]. PKCζ has also been reported to post-transcriptionally control the telomerase enzyme, through the phosphorylation of hTERT [291], thus contributing to a long-term proliferative potential of Ag-specific T cells through telomere elongation. The impact of PKC isoforms on T cell activity is summarized in Figure 1.

Among the multiple PKC isoforms expressed by B cells, PKCβI and PKCβII play a critical role in B cell development, activation/proliferation, and guide the microtubule polarization resulting from LFA-1 engagement in the IS [292,293,294,295]. PKCβ has also been reported to be implicated in either *IL2* gene transcription and/or release [295,296] in T cells, through the activation of MAPK/NF-κB/NFAT pathway, while at variance, another study only provided evidence of an involvement of PKCβ in IL-2 exocytosis. A PKCβ involvement in the increase in CD69 levels was involved in tissue residency [297] was also suggested, along with the production of CXCL8 chemokine [298], the latter reported as pro-tumorigenic in the TME by supporting tumor persistence, EMT, angiogenesis and impairment of anti-tumor responses [299].

### 4.2. Potential Impact of PKC on TME Features and Immunotherapies

#### 4.2.1. Impact of PKC on Intra-Tumor Macrophage Polarization

ICB agents work by reinvigorating anti-tumor T cell responses and have revolutionized cancer therapy showing unprecedented antitumor responses [300]. However, most patients do not respond adequately, especially to monotherapies. Therefore, a deeper knowledge of the mechanisms underlying resistance is strongly required. 

Favorable clinical responses to immunotherapy have been correlated with a highly infiltrated inflamed TME [301]. However, the degree of spontaneous immune infiltration in tumors varies extensively between individual patients; therefore, tailored patient-specific therapeutic manipulations can potentially improve the extent of intra-tumor T cell infiltration. Patients not responding to immunotherapy show high levels of circulating myeloid-derived suppressor cells (MDSCs), an immunosuppressive innate cell population which expands in pathological settings including cancer, comprising two subgroups: the polymorphonuclear MDSCs (PMN-MDSCs) and the monocytic MDSCs (M-MDSCs) [302]. 

According to a pre-print research report by Chaib et al. [303], PKCδ is highly expressed in mononuclear phagocytes involved in cancer growth. The absence of PKCδ in the preclinical models of knock-out mice was associated with reduced tumor development and a higher responsivity to PD-1 blockade. Moreover, the absence of PKCδ in M2-like macrophages has been related to higher T cell activation than in the presence of wild-type M2-like macrophages, delayed tumor growth and an immune landscape skewed towards a Th1 phenotype. Lastly, the loss of PKCδ has been shown to increase Ag presenting competence in macrophages and DCs and trigger type I and type II interferon signaling, proposing PKCδ that is a reasonable target to reprogram mononuclear phagocytes and increase intra-tumor T cell migration while improving the PD-1 blockade efficacy [303]. 

Similarly, in hepatocellular carcinoma (HCC) patients, a high expression of PKCα has been reported to stimulate the M2-like polarization of macrophages, accompanied by immune escape and a lack of response to the anti-PD1 blockade by promoting the nuclear import of ZFP64 [304]. 

At variance, the employment of a syngeneic mouse model has demonstrated how the pharmacological stimulation of PKC with the plant extract ingenol mebutate (PEP005) or prostratin PKC agonists is able to reduce the MDSC development from cellular precursors while stimulating the development of a cDC1-associated APC-like phenotype via the activation of p38MAPK [305], thus impairing the MDSC suppressive function. Of note, the combined activation of PKC and CD40 has been reported to significantly reduce breast cancer growth while increasing intra-tumor CD8^+^ T cell activation and T_RM_ frequency in a mouse preclinical model. This apparent discrepancy between the two studies from Chaib et al. [303,305] prompts the requirement for more specific PKC modulators, with either an inhibitory or agonistic effect, not able to target other PKC isoforms sharing structure resemblances, which can impair the expected outcomes. 

Among cancers poorly responding to ICB therapy, the EGFR–mutated lung tumor has been described to achieve a modest advantage in clinical settings [306,307], likely due to an insufficient intra-tumor T cell recruitment, speaking in favor of a non-inflamed tumor site. Small-molecule inhibitors of tyrosine kinase EGFR represent the favorite first-line approach for EGFR-mutant NSCLC. Nevertheless, frequent drug resistance severely restrains stable therapeutic effectiveness, although successful alternative approaches are not presently available. Of relevance, PKCδ represents one of the immune rheostats responsible for the establishment of non-inflamed phenotype in EGFR-mutated lung cancer, also by increasing the expression of PD-1 ligand PD-L1 on tumor cells [308], leading to immune exclusion and cancer escape from T cell surveillance. Accordingly, PKCδ blockade has been shown to improve intra-tumor T cell infiltration and inhibit tumor expansion while increasing tumor susceptibility to ICB therapy in both in vitro and in vivo settings. 

#### 4.2.2. Impact of PKC on Tumor-Infiltrating T Cells

While the normal function of PKCθ in T cells is well established, a deeper knowledge about its roles in immunotherapy resistance and dysfunctionality of T cells is strongly required. PKCθ can be endowed with both cytoplasmic and nuclear functions, and nuclear chromatin-associated PKCθ is increasingly acknowledged as pathogenic in cancer settings, while its cytoplasmic roles are relevant in normal T cell functions. Nuclear chromatin-associated is enriched in PKCθ in circulating tumor cells in patients with triple-negative breast cancer (TNBC) brain metastases and immunotherapy-resistant metastatic melanoma and has been associated with poor survival in immunotherapy-resistant disease. Furthermore, increased levels of nuclear PKCθ associated with the transcription factor ZEB1 have been detected within CD8^+^ T cells isolated from immunotherapy-resistant metastatic cancer patients. Based on the previous identification of a nuclear localization signal motif in PKCθ [309], a novel PKCθ inhibitor (nPKC-θi2) that specifically inhibits nuclear translocation of PKCθ without interrupting normal catalytic signaling in healthy T cells has recently been described [310]. The inhibition of nuclear PKCθ partially overcomes metastatic tumor growth by inhibiting mesenchymal signatures in murine TNBCs. In addition, nPKC-θi2 has been shown to impair the ZEB1/PKCθ complex while inducing cytokine release in CD8^+^ T cells isolated from immunotherapy-resistant patients. Overall, targeting nuclear PKCθ could represent a combinatorial approach able to re-invigorate dysfunctional T cells in immunotherapy-resistant patients.

Unfortunately, this could not be the case for some T cell NHL patients who have experienced hyper progression following PD-1 blockade, due to a significant re-invigoration of CD4^+^ malignant T cells expressing high levels of expressions of activated p-PKCθ (Thr538), leading to the hyper activation of the oncogenic TCR-associated pathway [311]. 

Lymphocyte activation gene 3 (LAG-3, also known as CD223) is an IC belonging to the immunoglobulin superfamily (IgSF) [312], strictly associated with human tumor prognosis, mostly negative, but also to positive outcomes, like in gastric carcinoma and melanoma [313]. LAG-3 is expressed by both effector T cells and Tregs [314] under continuous Ag stimulation, contributing to T cell exhaustion and increase in Treg immunosuppressive functions. Both the upregulation and the transport to the T cell surface of LAG-3 are mediated by PKC [315], although the distinctive kinase isoform involved is not specified in the study. While the reported PKC-mediated down-regulation of CD4 expression is mediated by a direct Ser phosphorylation and endocytosis [316,317], PKC may induce a conformational change of the adaptor molecule interacting with LAG-3, which leads to the IC translocation to the cell surface. This prompted the authors to propose that PKC signaling may act as one of the switches to turn off T cell activation to achieve T cell homeostasis.

According to the negative impact demonstrated by the seminal studies described above, a single-cell RNA sequencing (RNA-seq) study by Cron et al. [318] demonstrated that a single-nucleotide polymorphism (SNP) mutation, associated with a lower expression of PKCδ and implying a loss-of-function phenotype, results in a reduced B16.SIY melanoma growth, along with an increased intra-tumor infiltration of CD8^+^ T cells at the endpoint and better response to anti-PD-L1 therapy [318]. However, confirming previous studies, PKCδ loss has mostly been shown to modify gene expression in myeloid cell subsets, leading to the increased expression of M1-associated genes and the decreased expression of M2-associated genes in PKCδ^-/-^ chimeras. 

These findings establish that germline modifications in immune regulatory genes can profoundly affect anti-tumor immunity and the efficacy of PD-1/PD-L1 blockade, as described previously, in the case of decreased expression/activity of PKCδ in myeloid lineage mediating improved anti-tumor immunity by altering the M1/M2 ratio. 

Among other isoforms, PKCη has been shown to potentially synergize with CTLA-4 to mediate immune tolerance, by mediating the regulatory Treg function downstream of the checkpoint molecule [286], PKCη induces anti-inflammatory cytokine transcription by activating NF-κB downstream of the cascade [319]. Accordingly, the PKCη defect in murine models has been shown to reduce the tumor-suppressive activity of Tregs in the absence of autoimmune reactions [286] while reducing tumor growth [320]. 

Of note, a valuable study was conducted by Abdelatty et al. [321] by employing the single-cell gene set enrichment analysis and the ESTIMATE methods to analyze the tumor-infiltrating lymphocytes (TILs) and the immune components found within 28 cancer types, and correlating response to immunotherapies with PKC levels, based on human leukocytic antigen gene enrichment scores and PD-L1 expression. Univariate and multivariate Cox analysis was performed to evaluate the prognostic role of PKC genes in a pan-cancer analysis, and PKC isoenzymes were found to be solid biomarkers for the tumor immune status, with a distinctive PKC gene expression differently impacting the clinical outcome of cancer patients after immunotherapies. Altogether, the transcripts coding for PKCβ, PKCε and PKCθ were positively related to the response to immunotherapies, while PKCι and PKCζ proved mainly detrimental in most cancers.

Also, most PKC isoforms were directly related to intra-tumor PD-L1 expression, with the exception of PKCθ that instead showed a reversed correspondence with PD-L1 in 23 out of the 28 tumors analyzed. 

Accordingly, atypical PKCι has been shown to condition the tumor microenvironment via the YAP1 factor by reducing T cell infiltration and suppressing the host immune response in ovarian [322] and pancreatic adenocarcinoma (PDAC) [323]. Of note, PKCι-YAP1 signaling has been reported to increase the expression of PD-L1 in PDAC through STAT3 phosphorylation at Tyr705, contributing to PDAC resistance against cytotoxic NK cells [323].

Tumor-infiltrating CD8^+^ T cells expressing high levels of CD226, previously known as DNAX accessory molecule-1 (DNAM-1), possess greater self-renewal capacity and responsiveness [324]. PKC(ζ)-mediated phosphorylation of Ser329 [325] is critically required for CD226 adhesion and the association with lipid rafts and LFA [326], a prerequisite for phosphorylation at Tyr322-dependent full activation. Immune checkpoint T cell immunoreceptor with immunoglobulin and ITIM domains (TIGIT) affects CD8^+^ T and NK cell functionality [327] by engaging with CD226, similarly to the reported commitment between PD-1 and CD28 [224,225], and the blockade of TIGIT has been shown to improve CD8^+^ T cell responses selectively affecting CD226hiCD8^+^ T cells by promoting CD226 phosphorylation at Tyr322 [328]. Since CD226 agonist antibody-mediated activation of CD226 augments the effect of TIGIT blockade on CD8^+^ T cell responses, PKC can potentially represent a pharmacological target to stimulate intra-tumor CD8^+^ T cells to promote CD226 migration to the lipid raft to facilitate activation by phosphorylation at Tyr322.

As previously discussed, DGKζ facilitates DAG consumption, limiting PKC-dependent T cell activation and cytotoxic T cell responses. By reducing DAG consumption, the absence of DGKζ has been shown to improve the associated transcriptional programs, fostering IL-2 release and partly neutralizing the PD-1 repressive impact. In particular, DGKζ neutralization has been related to the decreased PD-1 expression, increase in cytotoxic CD8^+^ T cells, and reduced MC38 adenocarcinoma growth [329], outlining a task for DGKζ as a regulator of PD-1 expression.

Of note, the expression of PD-L1 has been associated with reduced aPKCλ levels and poor OS in cutaneous angiosarcoma (CAS) patients, along with the impaired phosphorylation of FoxO1 at Ser218 [330], which is mediated by the kinase [331]. Moreover, the inhibition of aPKCλ has been demonstrated to reduce the PD-L1 expression in cultured endothelial cells, suggesting that the combined treatment with ICIs and aPKCλ inhibitors could represent a therapeutic strategy for CAS patients.

**Figure 2:**
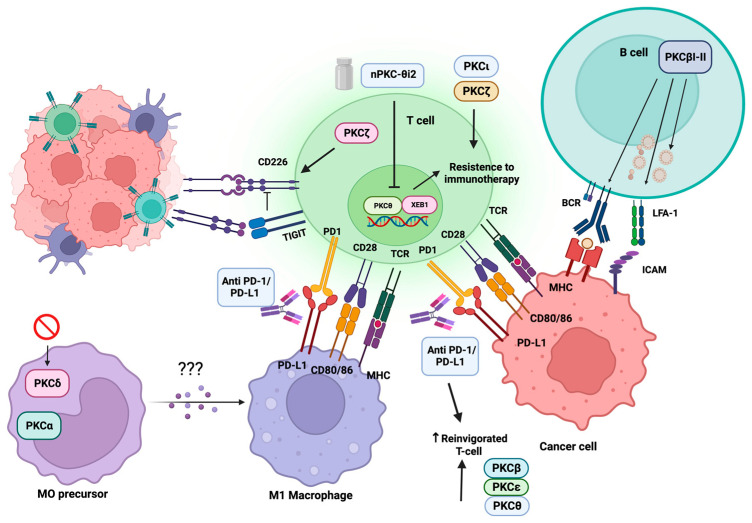
Effect of PKC on B cell activation and response to ICB. Effect of PKCβ on B cell activation and MTOC polarization; potential effects of PKCδ on intra-tumor macrophages polarization toward an M2 phenotype and response to ICB; negative impact of intra-nuclear PKCθ (via interaction with the chromatin) in terms of immunotherapy resistance: role of the nPKC-θi2 compound; impact of PKCζ on the activation of CD226, critical for competition with the inhibitory molecule TIGIT and for responsivity to TIGIT blockade; prognostic role of different PKC isoforms in terms of either immunotherapy response/resistance. Created with https://www.biorender.com (accessed on 28 June 2023).

The impact of the PKCβ on B cell functionality and the influence of PKC isoforms on the response to ICB are schematized in Figure 2.

## 5. Conclusions

Our focus on PKC isoforms, while underlining their prevalent oncogenic roles, also highlights controversial aspects mainly attributed to PKCδ and to the suppressor role of PKCζ. This picture is further complicated by the occurrence of mutations that can either increase or impair the activity of these kinases. Rare variants with significant clinical impact are reported by the ClinVar database. In particular, the mutation 501 of PKCδ is reportedly of uncertain significance (https://www.ncbi.nlm.nih.gov/clinvar/, accessed on 15 July 2023). An interesting PKCι mutation has been reported as pathogenic in SCLC [55], while of relevance, the R480C dPKCι mutation is one of the most detected in tumors [56]. Different alterations (i.e., single pint mutations, splicing variants, fusion proteins) can potentially represent useful markers for the diagnosis of specific tumors, in some cases even targetable with selective drugs, as in the case of enzastaurin, employed to treat melanoma and characterized by the presence of spliced PKCβ isoforms.

The complications and setbacks faced in the pharmacology of PKC have generated a difficult perception of the true impact of PKCs in the context of anticancer therapies. After almost three decades of clinical trials with PKC inhibitors, it is clear that the inhibition of PKC alone provides little or no clinical benefit in the treatment of cancer. Furthermore, most of these drugs have not only shown no significant clinical benefit, but in some cases have even worsened patient outcomes. Indeed, as reported by an extensive meta-analysis [332], the combination of enzastaurin [171,172] or aprinocarsen [203] with standard chemotherapy (pemetrexed, erlotinib, gemcitabine, cisplatin, carboplatin) has provided lower response rates and greater adverse effects as compared with chemotherapy alone in recurrent or malignant NSCLC patients [332].

In contrast, clinical studies with midostaurin plus standard chemotherapy in patients with FLT3-mutated AML [158], or with midostaurin alone in patients with advanced mast cell lymphoma [160] have shown significant clinical advantages, including improved overall response rates, longer event-free survival times and low toxicities. 

Whether these midostaurin-induced clinical benefits are due to tyrosine kinase inhibition alone or to the impairment of both, tyrosine kinase and PKC, needs to be evaluated by further investigations.

The failure of the pharmacological inhibition of PKCs in most clinical studies can be explained by the complex biological functions regulated by PKC isoenzymes and their significant expression heterogeneity in different types of cancer [333,334]. Whereas PKCs have generally been considered pro-oncogenic kinases, different isoenzymes are inactivated by loss-of-function mutations in a variety of cancers, thus supporting a potential tumor-suppressive rather than tumor-promoting role for distinctive PKC isoenzymes [334]. In fact, specific PKC isoforms have been implicated in various biological activities in tumor cells, including tumorigenic and anti-tumorigenic, anti-apoptotic or pro-apoptotic, proliferative or anti-proliferative activities [18,335]. Non-specific PKC isoenzymatic inhibitors, particularly those ATP-competitive, can thus block the activation of different PKC isoforms associated with both carcinogenic and antitumor effects, not leading to the expected therapeutic benefit.

A deeper understanding of the expression levels, the mutational status and of the role of distinct PKC isoforms in different cancers may aid in the identification of patients who may benefit from kinase-targeted therapies. Notably, the failure of aprinocarsen, which specifically targets PKCα, is not surprising, given the anti-growth/tumor-suppressive activity of PKCα in leukemia and colorectal, ovarian and possibly NSCL cancer models. Thus, a growing body of evidence suggests that the therapeutic exploitation of PKCα signaling in many cancers will require interventions that enhance rather than suppress PKCα function.

Although PKC inhibitors block the pro-proliferative and apoptotic functions of PKC, which are significantly activated in cancer settings, other cellular signals (e.g., AKT) that promote proliferation and are anti-apoptotic, may be able to replace the PKCs. Noteworthy, some inhibitors of PI3K, which is upstream of PKC and AKT, show significant clinical benefit in cancer treatment [336].

These considerations highlight the need for a better understanding of the pathways involved and the development of new PKC inhibitors for use in clinical practice, with higher isoenzyme specificity.

Furthermore, despite the current use of ICB agents to treat different cancers based on their ability to stimulate tumor-specific T cells, a substantial fraction of patients fails to respond adequately, thus supporting to the need for combined therapeutic strategies. Of note, mechanisms produced by the distinctive PKC isoforms described in this review have the potential to support or inhibit ICB-mediated immune recovery. Relevant results could potentially be achieved by inhibiting PKCδ in intratumoral myeloid cells, strongly biased towards the generation of immunosuppressive M2 macrophages. The resulting switch to a preferential M1 generation may ultimately lead to an increased intra-tumor CD8^+^ T cell infiltration [318], one of the prerequisites underlying the success of ICB therapy.

Furthermore, interesting compounds such as nPKC-θi2, by specifically targeting nuclear PKCθ without disturbing the normal catalytic signaling in T cells [310], can potentially help to prevent the development and metastatization of recalcitrant tumors such as TNBCs, while promoting CD8^+^ T cell function. Therefore, compounds of this type potentially represent promising therapeutic agents to be tested in combination with ICB in tumors that are otherwise poorly responsive to immunotherapies. Of note, the distinctive inhibition of specific forms of PKC with intracellular localizations linked to pathogenic states, may, at least in part, compensate for the current lack of isoform-selective inhibitors. 

We can therefore envision that a comprehensive examination of the molecular features of the tumor, in terms of either the expression of distinctive PKC isoenzymes or mutated forms, could contribute to improving the clinical outcome of the PKC blockade. This exploration could also suggest novel and feasible patient-specific immune landscapes featuring novel targets of PKC inhibition or stimulation. Indeed, individual PKC isoforms can also be potentially predictive of ICB response in several cancer types [321] or could be selectively modulated in immune cell subsets to promote antitumor responses and improve clinical outcomes after immunotherapies. Further investigations, aiming to better elucidate the interactions and the roles, either divergent or collaborative of the different isoforms in antitumor immune responses, may contribute to a better use of PKC both as a therapeutic target and as a biomarker of response to immunotherapies.

## Figures and Tables

**Table 1:** Mutations affecting PKC isoenzymes, functional loss, or new associated functions.

Isoenzyme	Mutation	Function	Type of Cancer/Pathologies
PKCα	D294G, located in the C2 domain	Kinase functional loss and inhibition of F-actin accumulation, thus interfering with the organization of cytoskeletal filaments at the cell–cell junctions [26]	Highly invasive pituitary and thyroid tumors [27]; adenomas (pituitary, follicular); thyroid carcinomas [28]
PKCα	D463H, affecting only one allele, present in heterozygosity	Affecting the highly conserved Asp residue, fundamental for the kinase activity, and leading to a different distribution and a reduced lifetime of the protein, likely favoring its phosphorylation [29]	Chordoid gliomas [30], affecting the third ventricle [29]
PKCα	M489V, coding SLC44A1-PKCα fusion protein	Gain-of-function is caused by the rearrangement between chromosomes 9 and 17, which generates a constitutive oncogenic and functional kinase [31,32]	Rare mixed neuronal-glial tumors known as PGNTs, difficult to diagnose, (e.g., papillary glioneuronal tumors) [32]; Alzheimer’s disease in mouse model [33]
PKCα, PKCβ and PKCδ	Gene fusion	Interaction with membrane-associated proteins, including podoplanin, CD63 and LAMTOR1 [34]	Benign fibrous histiocytoma [34]
PKCβII and PKCβI variants	Spliced isoforms with different C-terminal domain (V5 variants)	Specific maturation processing and distinctive cellular localization [35]; PKCβ loss correlates with melanin levels and oxidative stress response; decreased neocortical gene expression	Lung cancer cell lines [36]; melanoma [37]; autistic disorder [38]
PKCβII	P616A and P619A mutations	Abolish its maturation [39], the protein results unphosphorylated	Investigated in COS7 cells [39]
PKCβ	A509T mutation in heterozygosity affecting the C2 domain	Functional loss; hemizygous PKCβ has lower growth potential than when co-expressing A509T mutation; generation of dominant-negative with greater anchorage-independent cell proliferation [25]	Large intestine identified screening human colorectal cancer cell lines and a NCI-60 cell line panel [40,41];
PKCβ	D427N	Constitutively active open conformation able to increase NF-κB signaling [42]	Hematological malignancy [43]; T-cell leukemia-lymphoma [44] T-cell leukemia/lymphoma [45]; Sézary syndrome [46]
PKCγ	M501I	Change of the selectivity for the phosphorylation target in favor of a Thr, leading to the preferential recognition of different substrates, thus deviating the canonical kinase networks [47,48]	Lung cancer (dataset: TGCA—Cosmic Cured COSU417 (https://cancer.sanger.ac.uk/cosmic, accessed on 15 July 2023)
PKCδ	Changes in the DFG and APE motifs of the catalytic domain, such as the hinge region or in the Thr residue	Loss-of-function [49] with the inhibition of PKCδ, preventing the cleavage by caspase 3; this differently modulates their downstream targets, such as p53, leading to a decline of its transcription	Gastric cancer [50]; Autoimmune lymphoproliferative syndrome reported in https://www.ncbi.nlm.nih.gov/clinvar/ (accessed on 15 July 2023) with uncertain significance (NM_006254.4(PRKCD):c.1501G>A (p.Gly501Arg) and NM_006254.4(PRKCD):c.1501G>T (p.Gly501Trp))
PKCζ	K281W, dominant-negative PKCζ plasmid	In TRAMP-transfected cells, it reduces proliferation and enhances cell survival [51]; cell-polarizing deficit associated with multi-acinar structures and early luminal cell hyperproliferation [52]	Pancreatic islets and β-cells [53]; breast cancer [52]
PKCζ	Myristoylated PKC	This modification constitutively activates the kinase, increases cell proliferation and promotes apoptotic cell death [51]	TRAMP cell lines expressing FLAG-PKCζ-myr [51]
PKCι	Gene amplification, due to the association of *PRKCI* gene to the 3q26-29 amplicon, co-amplification with SOX2	Phox and Bem1 (PB1) binding domain of the protein of the 3q amplicon makes PKCι a therapeutic target [54]	Small-cell lung cancer [55]; pathogenic from https://www.ncbi.nlm.nih.gov/clinvar/ (accessed on 15 July 2023) (GRCh38/hg38 3q26.1-29(chr3:166137209-198125115)x3)
PKCι	R480C [56], previously mistakenly identified as R471C [57]	Arg471 is involved in the binding of lethal giant larvae 2; change of function: it modifies the recruitments of the corrected target substrates [57]	The most frequent mutation of PKCι in human cancer [56]

**Table 2:** Clinical outcomes of most relevant investigations analyzing PKC inhibitors in different cancer settings.

PKC Modulator and Structure/Sequence (https://pubchem.ncbi.nlm.nih.gov/docs/compounds, accessed on 17 July 2023)	Specificity	Inhibition Mechanism	Tumor Type	Phase of Study	Clinical Outcome	Ref.
UGN-01 7-hydroxystaurosporine 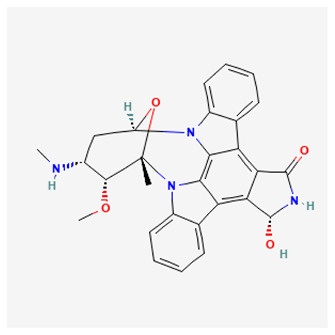	cPKC nPKC	Competitive with ATP binding site	Solid tumors Lymphoma Leukemia	I	Not relevant	[139,140,141,142,143,144,145] [141,146] [147,148]
Midostaurin (PKC412) N-benzoylstaurosporine 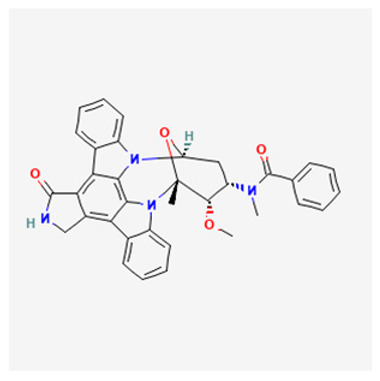	cPKC nPKC	Competitive with ATP binding site	Acute myeloid leukemia Melanoma Mutant FLT3-positive Acute Myeloid Leukemia	I, II, II IIA III	Survival benefit Favorable tolerability Not relevant Survival benefit Good tolerability	[155] [156] [158,159]
Sotrastaurin (AEB071) 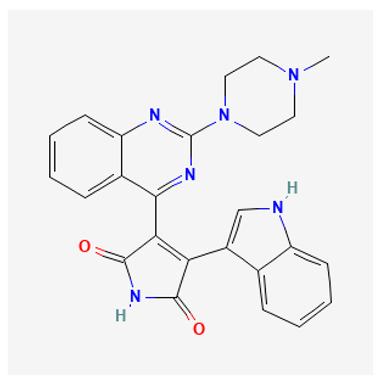	PKCα, β, θ, δ	Competitive with ATP binding site	Systemic mastocytosis Metastatic uveal melanoma	II I Ib	Survival benefit No unpredicted toxicities Favorable tolerability	[160] [166] [167]
Darovasertib (LXS196) 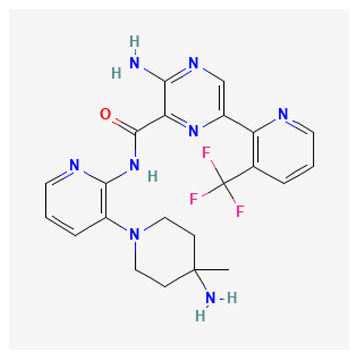	PKCα, PKCθ	Competitive with ATP binding site	Metastatic uveal melanoma	I	Favorable tolerability Promising clinical activity	[168]
Enzastaurin (LY317615) 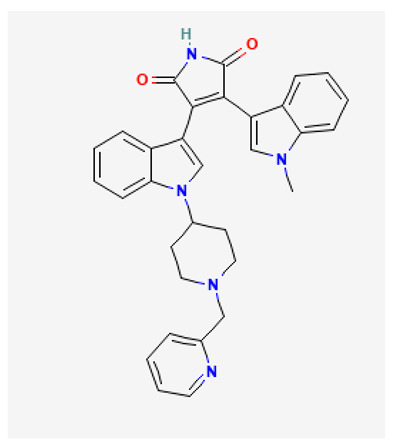	PKCβ	Competitive with ATP binding site	Solid tumors NSCLC Glioma Prostate cancer Ovarian cancer Multiple myeloma Brain metastasis Ovarian, peritoneal cancer Mantle cell lymphoma Breast cancer Cutaneous T cell lymphoma B cell lymphoma	I II II II II II II II II II II III	Favorable tolerability Not relevant Not relevant Not relevant Not relevant Not relevant Not relevant Not relevant Not relevant Not relevant Not relevant Clinical benefit when DGM1+	[170] [171,172] [173,174] [175] [176] [177] [178] [179] [180] [181] [182] [183]
Bryostatin 1 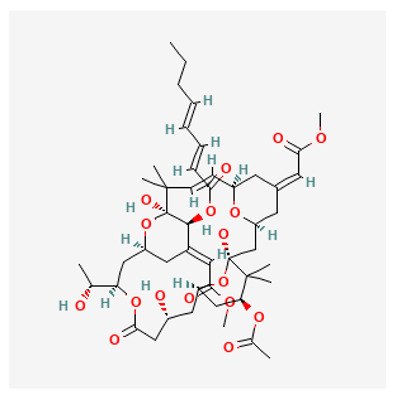	PKCα,ε,η	Competitive with phorbol ester binding site	Melanoma Renal carcinoma Colorectal cancer Non-Hodgkin’s lymphoma Multiple myeloma Head and neck cancer/sarcoma Cervical cancer Ovarian cancer Pancreatic cancer NSCLC Gastric/gastro- esophageal cancer Esophageal cancer Non-Hodgkin lymphoma	II II II II II II II II II II II II II	Not relevant Not relevant Not relevant Not relevant Not relevant Not relevant Not relevant Not relevant Not relevant Not relevant Clinical benefit Not relevant Clinical benefit in a subset of patients	[186] [187] [188] [189] [190] [191] [192] [193] [194] [195] [196] [197] [198]
Aprinocarsen (ISIS-3521/LY900003) 5′-GTTCTCGCTGGTGAGTTTCA-3′	PKCα	Antisense oligonucleotide binding to the 3′-UTR of human PKC-α mRNA	High-grade astrocytoma NSCLC Ovarian cancer Prostate cancer Breast cancer Colorectal cancer Non-Hodgkin’s Lymphoma	II III, II II II II II II	Not relevant clinical benefit Favorable tolerability Not relevant clinical benefit Favorable tolerability Not relevant clinical benefit Favorable tolerability Not relevant clinical benefit Favorable tolerability Not relevant clinical benefit Favorable tolerability Not relevant clinical benefit Favorable tolerability Not relevant clinical benefit Favorable tolerability	[202] [203] [204] [205] [206] [207] [208]

## Data Availability

Not applicable.
